# Genome Sequence of *Oxalobacter formigenes* Strain OXCC13

**DOI:** 10.1128/genomeA.00534-17

**Published:** 2017-07-13

**Authors:** Marguerite Hatch, Milton J. Allison, Fahong Yu, William Farmerie

**Affiliations:** a Department of Pathology, Immunology and Laboratory Medicine, University of Florida, College of Medicine, Gainesville, Florida, USA; b Iowa State University, Ames, Iowa, USA; c Interdisciplinary Center for Biotechnology Research, University of Florida, College of Medicine, Gainesville, Florida, USA

## Abstract

The lack of *Oxalobacter formigenes* colonization in the human gut is generally acknowledged as a risk factor for kidney stone formation since this microorganism can play an important role in oxalate homeostasis. Here, we present the genome sequence of OXCC13, a human strain isolated from an individual residing in Germany.

## GENOME ANNOUNCEMENT

*Oxalobacter formigenes*, an anaerobe with substrate specificity for oxalate, was isolated in 1985 and a new genus and species were established (1). This microorganism can play an important role in oxalate homeostasis, and the absence of this gut commensal is considered a risk factor in the formation of calcium oxalate kidney stones (2). The genomic sequence of *O. formigenes* strain OXCC13, determined by the Broad Institute Genome Sequencing Platform (RefSeq NZ_ACDQ00000000.1), is currently available. The present study was undertaken to determine the complete genome sequence of the OXCC13 strain, which has been archived in the Hatch laboratory since 2011 and is notated here as OXCC13^MH^.

A genomic DNA library was constructed following the protocol provided by Pacific Biosciences (Menlo Park, CA). Briefly, genomic DNA was sheared to an average fragment length of 20 kb by use of the SAGE ELF (Sage Science, Beverly, MA), end repaired, and then blunt-end ligated with single-molecule real-time (SMRT) bell oligonucleotide adaptors to construct a DNA fragment library for sequencing on the Pacific Biosciences RSII instrument. A single SMRT cell produced a total of 1.18 Gb in 71,943 polymerase reads having an *N*_50_ of 18.1 kb and a subread *N*_50_ of 11.5 kb.

The OxCC13^MH^ genome was assembled from long PacBio sequencing reads using HGAP version 3 (3). The OxCC13^M.H.^ genome was deposited in GenBank (accession number CP019430) and annotated using RAST (http://rast.nmpdr.org) (4–6). The complete OxCC13^MH^ genome contains a single contig of 2,433,653 bp and has an average GC content of 49.6%. A total of 2,599 genes were annotated by RAST, including 47 tRNAs, 6 ribosomal RNAs, and 2,499 predicted coding sequences (CDS). RAST annotation assigned 1,060 (43%) of the 2,499 OxCC13 CDS as members of 269 categorized subsystems. Subsystems are defined as sets of functional roles implementing specific biological process or structures (7). In general, subsystems are considered biological pathways. The most abundant subsystem classifications included 202 genes involved in protein metabolism; 169 involved in metabolism of cofactors, vitamins, prosthetic groups, and pigments; 205 involved in amino acid and derivative metabolism; and 108 involved in carbohydrate metabolism. A total of 1,439 CDS (57%) were not assigned to specific subsystems.

The complete, annotated OxCC13^MH^ genome was compared to *O. formigenes* CC13 (NCBI RefSeq NZ_ACDQ00000000.1). The OxCC13^MH^ genome was assembled as a single closed circular sequence of 2,433,653 bp. The public OxCC13 draft sequence (RefSeq NZ_ACDQ00000000.1) contains 9 scaffolds with a total length of 2,436,766 bp. The largest scaffold (NCBI accession number GG658170.1) is 2,424,267 bp. At the protein level, 2,447 of 2,499 (98%) OxCC13^MH^ CDS have greater than 99% amino acid identity with CDS identified in OxCC13. MUMmer (https://sourceforge.net/projects/mummer/) alignment of OxCC13^MH^ with OxCC13 showed the genome sequences to be collinear, with a collective total of approximately 4.5 kb of structural variation, mostly in the form of small deletions, tandem expansions, and a small repeat contraction. Besides the small structural variations, at the nucleotide level, there is less than 0.03% difference between genomes. The overall homology between OxCC13^MH^ and OxCC13 suggests, as expected, that both genome sequences are derived from the same genomic source DNA.

### Accession number(s).

This genome sequencing project was deposited in GenBank under accession number CP019430.

## References

[1] Allison MJ, Dawson KA, Mayberry WR, Foss JG 1985 *Oxalobacter formigenes* gen. nov., sp. nov.: oxalate-degrading anaerobes that inhabit the gastrointestinal tract. Arch Microbiol 141:1–7. doi:10.1007/BF00446731. 3994481

[2] Hatch M, Freel RW 2008 The roles and mechanisms of intestinal oxalate transport in oxalate homeostasis. Semin Nephrol 28:143–151. doi:10.1016/j.semnephrol.2008.01.007. 18359395 PMC2430047

[3] Chin CS, Alexander DH, Marks P, Klammer AA, Drake J, Heiner C, Clum A, Copeland A, Huddleston J, Eichler EE, Turner SW, Korlach J 2013 Nonhybrid, finished microbial genome assemblies from long-read SMRT sequencing data. Nat Methods 10:563–569. doi:10.1038/nmeth.2474. 23644548

[4] Overbeek R, Olson R, Pusch GD, Olsen GJ, Davis JJ, Disz T, Edwards RA, Gerdes S, Parrello B, Shukla M, Vonstein V, Wattam AR, Xia F, Stevens R 2014 The SEED and the Rapid Annotation of microbial genomes using Subsystems Technology (RAST). Nucleic Acids Res 42:D206–D214. doi:10.1093/nar/gkt1226. 24293654 PMC3965101

[5] Brettin T, Davis JJ, Disz T, Edwards RA, Gerdes S, Olsen GJ, Olson R, Overbeek R, Parrello B, Pusch GD, Shukla M, Thomason JA III, Stevens R, Vonstein V, Wattam AR, Xia F 2015 RASTtk: a modular and extensible implementation of the RAST algorithm for building custom annotation pipelines and annotating batches of genomes. Sci Rep 5:8365. doi:10.1038/srep08365. 25666585 PMC4322359

[6] Aziz RK, Bartels D, Best AA, DeJongh M, Disz T, Edwards RA, Formsma K, Gerdes S, Glass EM, Kubal M, Meyer F, Olsen GJ, Olson R, Osterman AL, Overbeek RA, McNeil LK, Paarmann D, Paczian T, Parrello B, Pusch GD, Reich C, Stevens R, Vassieva O, Vonstein V, Wilke A, Zagnitko O 2008 The RAST Server: rapid annotations using subsystems technology. BMC Genomics 9:75. doi:10.1186/1471-2164-9-75. 18261238 PMC2265698

[7] Overbeek R, Begley T, Butler RM, Choudhuri JV, Chuang HY, Cohoon M, de Crécy-Lagard V, Diaz N, Disz T, Edwards R, Fonstein M, Frank ED, Gerdes S, Glass EM, Goesmann A, Hanson A, Iwata-Reuyl D, Jensen R, Jamshidi N, Krause L, Kubal M, Larsen N, Linke B, McHardy AC, Meyer F, Neuweger H, Olsen G, Olson R, Osterman A, Portnoy V, Pusch GD, Rodionov DA, Rückert C, Steiner J, Stevens R, Thiele I, Vassieva O, Ye Y, Zagnitko O, Vonstein V 2005 The subsystems approach to genome annotation and its use in the project to annotate 1000 genomes. Nucleic Acids Res 33:5691–5702. doi:10.1093/nar/gki866. 16214803 PMC1251668

